# Epidemiology and outcomes of acute kidney injury in elderly chinese patients: a subgroup analysis from the EACH study

**DOI:** 10.1186/s12882-016-0351-2

**Published:** 2016-09-29

**Authors:** Shuwang Ge, Sheng Nie, Zhangsuo Liu, Chunbo Chen, Yan Zha, Jing Qian, Bicheng Liu, Siyuan Teng, Anping Xu, Wei Bin, Xin Xu, Gang Xu

**Affiliations:** 1 Department of Nephrology, Tongji Hospital, Tongji Medical College, Huazhong University of Science and Technology, 1095 Jiefang Ave, Wuhan, China; 2 National Clinical Research Center for Kidney Disease, State Key Laboratory of Organ Failure Research, Nanfang Hospital, Southern Medical University, Guangzhou, China; 3 The First Affiliated Hospital of Zhengzhou University, Zhengzhou University, Zhengzhou, China; 4 Guangdong General Hospital, Guangzhou, China; 5 Guizhou Provincial People’s Hospital, Guiyang Medical University, Guiyang, China; 6 Huashan Hospital, Fudan University, Shanghai, China; 7 Zhongda Hospital, Southeast University, Nanjing, China; 8 The Second Affiliated Hospital of Dalian Medical University, Dalian Medical University, Dalian, China; 9 Sun Yat-Sen Memorial Hospital, Sun Yat-Sen University, Guangzhou, China

**Keywords:** Acute kidney injury, Elderly, Chinese, Epidemiology, Outcomes

## Abstract

**Background:**

Information on acute kidney injury (AKI) in elderly hospitalized patients is limited. This study aims to assess the incidence, risk factors and outcomes of AKI in elderly Chinese patients.

**Method:**

The Epidemiology of AKI in Chinese Hospitalized adults (EACH) study is a multicenter, retrospective cohort study conducted in nine regional central hospitals across China. Patients aged more than 65 years were selected from the EACH study for this analysis. A novel approach with adjustment for frequency of serum creatinine was used to estimate the incidence of AKI in elderly patients. In-hospital outcomes, including mortality, renal recovery, length of stay and daily cost of elderly patients, were analyzed and compared with outcomes in younger patients.

**Results:**

Of 144,232 adult patients in the EACH study, 42,737 (29.63 %) patients were 65 years or older, including 9773 very elderly patients (≥80 years old). The incidence of AKI was 15.44 % in patients 65–79 years old (community-acquired (CA) AKI of 3.89 % and hospital-acquired (HA) AKI of 11.55 %) and 22.22 % in the very elderly group (CA-AKI of 6.58 % and HA-AKI of 15.64 %). The mortality rate of AKI was 10.3 % in patients aged from 65 to 80 and 19.6 % in patients older than 80 years. AKI incidence, in-hospital mortality, percentage of patients requiring dialysis and percentage without renal recovery were higher in elderly patients than in younger patients.

**Conclusion:**

The incidence of AKI in elderly Chinese hospitalized patients is high, which becomes a substantial burden on medical care in China.

## Background

Acute kidney injury (AKI) is a common disorder, characterized by an abrupt or rapid decline in renal filtration function. The incidence of dialysis-dependent AKI increased by 10 % every year in the past decade [1]. However, the incidence of AKI in hospitalized patients varied in different studies, depending on the definition of AKI, frequency of serum creatinine (SCr) tests, clinical setting of study population, and economical level of countries [2–4]. In the Epidemiology of AKI in Chinese Hospitalized adults (EACH) study, by using a novel approach with adjustment for the frequency of SCr tests and other potential confounders, we have demonstrated that the incidence of AKI is 11.6 % in China, the largest developing country with 20 % of the world’s population [5]. Of note, the detection rate of AKI was only 0.99 % by KDIGO criteria without adjustment for the frequency of SCr tests in another cross-section study and under-diagnosis and under-treatment rates of AKI in China are extremely high [6], which may lead to poor outcomes for patients.

Elderly people have aging kidneys undergoing structural and functional changes that decrease auto regulatory capacity and increase susceptibility to damage [7]. The incidence rate of AKI is higher in the elderly population than in younger populations, and age is a major predictive factor of mortality in patients with AKI [8]. In addition to age-related changes in the kidney, multiple chronic comorbidities (chronic kidney disease, cardiovascular disease, diabetes and sepsis), exposure to nephrotoxic medications, oxidative stress, hypovolemia and surgery may account for the increased risk of developing AKI in elderly hospitalized patients [9]. It has been demonstrated that there is a significantly lower recovery rate of kidney function in elderly patients than in younger patients [10].

With the development of society and economy, life expectancy in China has increased to 73.5 years for males and 79.9 years for females according to WHO data published in 2013 [11]. Currently, nearly one in ten Chinese is aged over 65, and this number will increase to one in four by 2050, which will be a heavy burden on society. However, there are few studies about AKI in the elderly Chinese population [12–15]. In addition, several single-center studies did not adjust the frequency of SCr tests, which may have led to underestimation of the incidence of AKI in hospitalized elderly Chinese patients [14, 15].

In this large retrospective cohort study of hospitalized adults in China, we aimed to demonstrate the incidence rate, risk factors and in-hospital outcomes of AKI in elderly patients by using a novel analytical method to minimize the impact of frequency of SCr tests.

## Methods

### Study design, setting and participants

A multicenter, retrospective cohort study (the EACH study) was previously conducted [5]. Patients admitted between January 1 and December 31, 2013 from nine regional central hospitals across Northern, Central, and Southern China were enrolled in the EACH study. Patients with a history of stages 4–5 chronic kidney disease (CKD), maintenance dialysis or renal transplantation were excluded. Patients with less than two SCr tests in a 7-day window during their first 30 days of hospitalization were also excluded. Elderly patients (aged more than 65 years) were selected from the cohort for current analysis. The study protocol was approved by the Medical Ethics Committee of Nanfang Hospital (No. NFEC-2014-098). This study has adhered to the STROBE guidelines on reporting of cross-sectional data.

### Data sources

Clinical data including patient age, gender, admission and discharge data, SCr values and test frequency, medication, surgical procedures and dates, in-hospital death, and total cost were collected from electronic hospital and laboratory databases. Nephrotoxic drug exposure and other risk factors were assessed by trained nephrologists. The study protocol was approved by the medical ethics committee of Nanfang Hospital.

### Identification and classification of AKI

AKI was defined as an increase in SCr by 0.3 mg/dl within 48 h or a 50 % increase in SCr from the baseline within 7 days according to the KDIGO criteria [16]. Patients were classified according to the KDIGO criteria as specified by stage 1, stage 2 and stage 3. The date of AKI onset was defined as the earliest day that the SCr change met the KDIGO criteria. The stage of AKI was determined using the peak SCr level after the date of AKI onset.

Community-acquired (CA) AKI was defined in the clinical setting as meeting at least one of the following criteria: (1) Patients were diagnosed with AKI on admission according to diagnosis code; (2) SCr change during the first day of admission met the KDIGO definition; (3) SCr on admission was ≥1.4 mg/dl in men or ≥1.1 mg/dl in women and ≥1.5-fold of the baseline SCr level. The baseline SCr was defined as the lowest SCr during hospitalization for CA-AKI.

Hospital-acquired (HA) AKI was defined as AKI patients who did not meet the CA-AKI criteria. The baseline SCr was defined as the mean of SCr levels within the 7 days before AKI onset for HA-AKI.

### Definition of outcomes

Patient outcomes included in-hospital death, renal recovery, requirement for RRT, length of stay (LOS) in hospital and daily cost of hospitalization. Renal recovery of AKI was defined as a decreasing SCr to within the non-AKI range and at least 0.3 mg/dl below the peak level without RRT.

### Determination of comorbidity

The presence of comorbidities was identified by diagnosis codes at admission or past history in medical record. The Charlson comorbidity score was used to assess the burden of comorbidity.

### Statistical analysis

Statistical analyses were performed using R software, version 3.1.1, and the survival package, version 2.37, with statistical significance set at *P* < 0.05. Categorical variables were summarized as number and percentage, and continuous variables were expressed as the mean ± standard deviation and median with interquartile range (skewed distribution). One-way ANOVA was used for continuous variables, and a *X*^2^ test was used for categorical variables. Incidence of AKI was calculated by a novel approach with adjustment for the frequency of SCr tests [5]. Risk factors were determined by Cox proportional hazard model. Population attributable fractions (PAF) were used to assess the contribution of different risk factors to AKI. Cumulative rates of in-hospital death in the subgroups were determined using the Kaplan–Meier method by AKI status, and corresponding HRs were calculated using the Cox proportional hazard model with adjustment for age, sex, comorbidities, and clinical procedures.

## Results

Of 144,232 patients aged over 18 years included in the EACH cohort study, 42,737 (29.63 %) patients were 65 years or older, including 9773 (6.8 %) very elderly patients (≥80 years old). Characteristics of patients by age category are provided in Table 1. Compared with the younger group, elderly patients were more often male and had a higher prevalence of CKD, especially in patients older than 80 years. In addition, the Charlson comorbidity index scores in the elderly group were higher than in the younger group. The elderly group had a much higher number and frequency of SCr tests, which may lead to more frequent detection of AKI (Table 1).

**Table 1:** Demographic and clinical characteristics of patients by age category

	Non-AKI				AKI			
Characteristic	18–64 years (*n* = 92391)	65–79 years (*n* = 28934)	≥80 year (*n* = 8061)	*P* value	18–64 years (*n* = 9104)	65–79 years (*n* = 4030)	≥80 year (*n* = 1712)	*P* value
Age (yr)	46.55 ± 12.43	71.33 ± 4.32	84.14 ± 3.64	<0.001	47.41 ± 12.34	71.76 ± 4.39	84.54 ± 3.71	<0.001
Male (%)	52.7	59.1	61.9	<0.001	59.5	60.7	61.1	0.25
Geographic location (%)				<0.001				<0.001
Northern	26.1	27.9	25.1		28	28.2	25.3	
Central	34	29.7	32.8		28.8	28.9	34.6	
Southern	39.9	42.3	42.1		43.2	42.9	40.1	
CKD (%)	3.2	12.5	26.1	<0.001	17.4	31.7	42.5	<0.001
Charlson comorbidity index	1.17 ± 1.32	1.56 ± 1.46	1.87 ± 1.65	<0.001	1.35 ± 1.44	1.92 ± 1.65	2.28 ± 1.8	<0.001
BaselineSCr (umol/L)	66.16 ± 23.49	76.82 ± 27.19	84.91 ± 29.44	<0.001	77.33 ± 41.48	84.26 ± 40.52	87.26 ± 36.18	<0.001
Peak SCr (umol/L)	—	—	—	—	180.1 ± 177.35	190.29 ± 170.84	198.31 ± 167.49	<0.001
No. of SCr tests	3.43 ± 2.48	3.4 ± 2.43	3.62 ± 2.96	<0.001	7.81 ± 8.11	7.65 ± 7.52	9.14 ± 13.44	<0.001
Frequency of SCr test^a^	0.39 ± 0.23	0.43 ± 0.26	0.43 ± 0.26	<0.001	0.49 ± 0.26	0.5 ± 0.27	0.53 ± 0.27	<0.001
AKI Subtypes (%)^b^				—				<0.001
CA-AKI	—	—	—		2.76	3.89	6.58	
HA-AKI	—	—	—		8.78	11.55	15.64	
AKI stage (%)				—				<0.001
1	—	—	—		56.6	60.7	59.4	
2	—	—	—		20.1	20.4	21.2	
3	—	—	—		23.3	18.9	19.4	
ICU (%)	8.1	8.8	9.2	<0.001	31.3	31.7	29.2	0.19

Length of stay and daily cost are presented in median (25th, 75th percentile). Values expressed with a ± sign are presented as the mean ± SD

*SCr* serum creatinine, *CNY* Chinese yuan, *CA* community-acquired, *HA* hospital-acquired

^a^Defined as number of days with SCr tests divided by length of stay

^b^Adjusted for age, sex, comorbidities and frequency of SCr test

### Incidence of AKI

The incidence of AKI adjusted for the frequency of SCr tests was 15.44 % in patients 65–80 years old (CA-AKI of 3.89 % and HA-AKI of 11.55 %) and 22.22 % in the very elderly group (CA-AKI of 6.58 % and HA-AKI of 15.64 %), whereas in the younger group, the incidence of AKI was much lower (CA-AKI of 2.76 % and HA-AKI of 8.78 %) (Table 1). In addition, there is a significant correlation between AKI stage and the age of patients. We identified a higher rate of AKI stage 1 and a lower rate of AKI stage 3 in patients 65–80 year old (Table 1).

### Risk factors for AKI

In patients older than 65 years, a significant association between AKI incidence and age was observed in CA-AKI groups (Table 2). Male patients were at increased risk for AKI compared to female patients, especially for HA-AKI. The PAF of the risk factors was used to assess their contribution to AKI in elderly patients. The top three risk factors for HA-AKI, ranked in order of decreasing PAF, were CKD, intensive care and heart failure. CKD, pneumonia and shock were the top three risk factors for CA-AKI. Of note, CKD was a major contributor to both HA-AKI (mean PAF of 24.8) and CA-AKI (mean PAF of 26.5) (Table 2). The PAF of CKD in patients older than 80 years is 30.7, which is much higher than the PAF of 10.4 in younger groups, suggesting CKD is an essential risk factor for elderly patients (Table 3).

**Table 2:** Risk factors for community- and hospital-acquired AKI in elderly patients

	HA-AKI			CA-AKI		
Risk factors	Frequency (%)	OR (95%CI)^a^	PAF	Frequency (%)	OR (95%CI)^a^	PAF
Age						
65-69 years		Reference			Reference	
70-74 years		0.99 (0.9 to 1.09)			1.17 (1.01 to 1.37)	
75-79 years		1.13 (1.02 to 1.24)			1.25 (1.07 to 1.47)	
>80 year		1.1 (0.99 to 1.21)			1.68 (1.44 to 1.95)	
Sex						
Male		1.19 (1.1 to 1.29)			1.06 (0.94 to 1.2)	
Comorbidity						
CKD	15.5	4.62 (4 to 5.33)	24.8	16.4	4.85 (3.93 to 6)	26.5
Heart failure	15.3	1.56 (1.42 to 1.72)	8.8	15.7	1.2 (1.03 to 1.4)	2.8
CAD	26.1	1.28 (1.18 to 1.39)	6.4	26.5	0.86 (0.76 to 0.98)	
Shock	1.3	2.05 (1.77 to 2.37)	3.7	1.6	4.57 (3.64 to 5.74)	6
Acute myocardial infarction	3.1	1.72 (1.48 to 2.01)	2.7	3.2	1.46 (1.11 to 1.92)	1.4
Stroke	13.5	1.17 (1.06 to 1.28)	2.6	13.3	1 (0.86 to 1.17)	
Pneumonia	14	1.08 (0.99 to 1.18)	1.8	14.4	1.8 (1.58 to 2.05)	11.1
Gastrointestinal bleeding	2.2	1.33 (1.12 to 1.57)	1	2.3	1.59 (1.22 to 2.07)	1.5
Liver disease	2.3	1.37 (1.11 to 1.68)	0.7	2.3	1.2 (0.86 to 1.67)	0.4
Sepsis	0.6	1.25 (1 to 1.57)	0.5	0.8	2.55 (1.8 to 3.6)	1.6
Trauma	1	1.45 (1.12 to 1.87)	0.5	1	0.85 (0.51 to 1.41)	-
Diabetes	18.6	1.02 (0.93 to 1.11)	0.3	18.9	1.11 (0.96 to 1.27)	1.8
Hematologic malignancy	2.2	1.14 (0.91 to 1.42)	0.3	2.3	1.44 (1.06 to 1.97)	0.8
Urinary tract obstruction	1.3	1.14 (0.86 to 1.51)	0.2	1.5	4.39 (3.41 to 5.66)	3.9
Burn	0	0.61 (0.09 to 4.34)		0	6.3 (1.37 to 28.99)	0.1
Rheumatic disease	1.5	1.01 (0.78 to 1.31)		1.5	1.15 (0.78 to 1.69)	0.2
Renal disease	2.1	0.93 (0.74 to 1.16)		2.3	1.88 (1.46 to 2.42)	2.2
tuberculosis	0.5	0.56 (0.29 to 1.09)		0.5	0.73 (0.29 to 1.8)	
COPD	6	0.95 (0.82 to 1.09)		6.1	1.09 (0.89 to 1.33)	
Solid cancer	25.8	0.92 (0.85 to 1)		24.8	0.61 (0.52 to 0.71)	
Hypertension	39.1	0.93 (0.86 to 1.01)		39.3	0.94 (0.85 to 1.04)	
Clinical setting for HA-AKI						
Intensive care	11.3	2.42 (2.22 to 2.63)	19.4			
Non-cardiac operation	45.1	1.17 (1.08 to 1.28)	6.8			
Cardiac operation	1.3	2.19 (1.84 to 2.6)	3.8			
Intervention operation	16.6	1.29 (1.17 to 1.43)	3.8			

*HA-AKI* hospital-acquired AKI, *CA-AKI* community-acquired AKI, *OR* odds ratio, *95 % CI* 95 % confidence interval, *PAF* population attributable fraction, *CAD* coronary artery disease; Intensive care: Stay in ICU (intensive care unit) during hospitalization

^a^Adjusted for age, sex, comorbidities and clinical procedures

**Table 3:** Risk factors for AKI patients by age category

Risk factors	18–64 years			65–79 years			≥80 year		
	%	OR (95 % CI)^a^	PAF	%	OR (95 % CI)^a^	PAF	%	OR (95 % CI)^a^	PAF
Sex									
Male	53.2	1.3 (1.22 to 1.38)			1.33 (1.21 to 1.45)			1.44 (1.25 to 1.66)	
Comorbidity									
CKD	4.1	4.31 (3.77 to 4.92)	10.4	13.2	5.64 (4.76 to 6.68)	21.6	26.7	6.89 (5.39 to 8.8)	30.7
Heart failure	5.4	3 (2.75 to 3.28)	6.9	13.8	1.86 (1.66 to 2.09)	7.5	21.2	1.4 (1.2 to 1.65)	5.7
Pneumonia	7.2	1.73 (1.6 to 1.86)	4.6	11.8	1.84 (1.66 to 2.04)	7.2	24.3	1.83 (1.6 to 2.09)	11.9
Shock	0.9	6.05 (5.19 to 7.05)	3.1	1.3	7.61 (6.07 to 9.56)	4.2	2.6	7.24 (5.3 to 9.89)	5.7
Stroke	5.2	1.4 (1.27 to 1.54)	1.7	11.7	1.28 (1.14 to 1.43)	2.4	19.9	1.2 (1.03 to 1.4)	2.7
Urinary tract obstruction	2.4	1.67 (1.47 to 1.9)	1.4	1.7	2.16 (1.71 to 2.72)	1.3	1.1	2.75 (1.72 to 4.39)	1
Liver disease	3.7	1.3 (1.16 to 1.46)	0.9	2.6	1.18 (0.94 to 1.47)	0.3	1.5	1.85 (1.21 to 2.84)	0.8
Sepsis	0.7	2.55 (2.08 to 3.12)	0.9	0.7	2.8 (2.01 to 3.9)	0.9	0.9	6.55 (3.9 to 11.02)	1.7
Gastrointestinal bleeding	1.5	1.49 (1.28 to 1.75)	0.7	2.1	1.49 (1.2 to 1.85)	0.9	3.2	1.72 (1.28 to 2.31)	1.5
Trauma	2.6	1.34 (1.17 to 1.53)	0.7	1	1.56 (1.15 to 2.12)	0.5	1	1.56 (0.89 to 2.73)	0.3
Acute myocardial infarction	1.3	1.82 (1.46 to 2.25)	0.6	3.1	2.04 (1.68 to 2.48)	2.1	3.5	2.18 (1.63 to 2.91)	2.4
Diabetes	8	1.08 (1 to 1.17)	0.6	17.9	0.94 (0.85 to 1.05)		21.6	0.9 (0.77 to 1.05)	
Hypertension	13.3	1.02 (0.9 to 1.15)	0.3	36.2	0.88 (0.82 to 0.95)		48.9	0.94 (0.82 to 1.08)	
Rheumatic disease	2.5	1.14 (1 to 1.31)	0.3	1.6	1.09 (0.83 to 1.45)	0.1	1.2	1.25 (0.74 to 2.1)	0.2
Hematologic malignancy	3.3	1.09 (0.95 to 1.24)	0.2	2.6	1.07 (0.85 to 1.35)	0.1	1.1	2.04 (1.26 to 3.29)	0.7
Burn	0.1	1.71 (1.04 to 2.82)	0.1		1.6 (0.33 to 7.73)		0	4.76 (0.42 to 54.08)	
Renal disease	2.8	1.03 (0.9 to 1.16)	0.1	2.2	1 (0.8 to 1.25)		2.6	1.48 (1.05 to 2.09)	0.9
COPD	0.6	0.61 (0.44 to 0.84)		4.6	0.92 (0.77 to 1.09)		11	1.08 (0.89 to 1.31)	0.7
Tuberculosis	0.7	0.36 (0.24 to 0.55)		0.5	0.67 (0.37 to 1.25)		0.4	0.41 (0.1 to 1.76)	
CAD	7.7	0.81 (0.74 to 0.89)		24	0.98 (0.89 to 1.07)		32.9	1.06 (0.91 to 1.23)	1.3
Solid cancer	28.8	0.8 (0.75 to 0.85)		27.5	1.06 (0.96 to 1.16)	1.1	17.9	1.15 (0.98 to 1.36)	1.7

*HA-AKI* hospital-acquired AKI, *CA-AKI* community-acquired AKI, *OR* odds ratio, *95 % CI* 95 % confidence interval, *PAF* population attributable fraction, *CAD* coronary artery disease

^a^Adjusted for age, sex, comorbidities and clinical procedures

Elderly patients were more likely to be exposed to nephrotoxic drugs. Our data demonstrated that 38.6 % of AKI cases in 65–80 years old group and 51.4 % in the very elderly group were possibly drug induced, which is much higher than 32.4 % of AKI cases in patients aged 18–64 years (Table 4). Renin angiotensin system inhibitors and diuretics were not included in nephrotoxic drug list in this study because they mainly impact on renal perfusion. Of note, the use of Chinese traditional medicine/remedies and NSAIDs were very common in elderly patients, especially in patients older than 80 years. More than one-third of very elderly patients had a history of exposure to Chinese traditional medicine/remedies before AKI onset (Table 4).

**Table 4:** Percentage of drug induced AKI cases by age category

	18–64 years (%)	65–79 years (%)	≥80 year (%)	*P* value
Nephrotoxic drugs	32.4	38.6	51.4	<0.001
Chinese traditional medicine/herbs	27.8	30.5	33.8	<0.001
NSAIDs	18.5	26.8	29.2	<0.001
Antibiotics	17.5	13.8	15.1	<0.001
Contrast media	6.1	8.3	7.7	<0.001
Anticancer drugs	4.8	2.7	0.7	<0.001

*NSAIDs* nonsteroidal anti-inflammatory drugs

### In-hospital outcomes mortality

The mortality rate of AKI was 6.2 % in the young patient group, 10.3 % in patients aged 65 to 80 and 19.6 % in patients older than 80 years (Table 5). There was a significant association between mortality from AKI and the severity of AKI in elderly patients. The HRs of in-hospital death adjusted for age, sex, comorbidities, and surgical procedures were 7.1, 15.2 and 29.7, respectively, for stage 1, stage 2 and stage 3 of AKI (Table 6).

**Table 5:** Outcome of patients by age category

Outcome	18–64 years (%)		65–79 years (%)		≥80 year (%)		*P* value
	Non-AKI	AKI	Non-AKI	AKI	Non-AKI	AKI	
	(*n* = 92391)	(*n* = 9104)	(*n* = 28934)	(*n* = 4030)	(*n* = 8061)	(*n* = 1712)	
Death (%)							
All	371 (0.4)	561 (6.2)	251 (0.9)	416 (10.3)	173 (2.1)	336 (19.6)	<0.001
AKI with dialysis	—	14.8	—	35.7	—	52.6	<0.001
Dialysis (%)	—	371 (5.6)	—	129 (4.5)	—	57 (5)	0.086
Recovery of HA-AKI (%)	—	2684 (41.7 %)	—	1098 (39.1 %)	—	373 (33.8 %)	<0.001
Length of stay (d)	13 (8,20)	17 (11,27)	13 (8,19)	17 (11,26)	14 (9,20)	17 (11,29)	<0.001
Daily cost (CNY)	1752 (1052,3018)	2621 (1426,4636)	1673 (1047,3096)	2541 (1398,4462)	1510 (2542,1012)	2190 (1338,3863)	<0.001

Length of stay and daily cost are presented in median (25th, 75th percentile)

The other cells are expressed as N (% within age and AKI strata)

*HA-AKI* hospital-acquired AKI, *CNY* Chinese yuan

**Table 6:** Outcome of elderly patients by AKI stages

Outcome	S0 (*n* = 36995)	S1 (*n* = 3457)	S2 (*n* = 1187)	S3 (*n* = 1098)	*P* value
Structure of AKI					<0.001
CA-AKI	—	24.7	44.1	41.4	
HA-AKI	—	75.3	55.9	58.6	
Death (%)	1.1	7.1	15.2	29.7	<0.001
Dialysis (%)	—	—	—	25.9	—
Recovery of HA-AKI (%)	—	42.3	33.6	22.6	<0.001
Length of stay(d)	13 (9,19)	16 (10,24)	18 (11,28)	19 (11,33)	<0.001
Daily cost (CNY)	1630 (1038,2944)	2173 (1282,3872)	2534 (1481,4168)	3450 (1798,6035)	<0.001

Length of stay and daily cost are presented in median (25th, 75th percentile)

The other cells are expressed as N (% within age and AKI strata)

*HA-AKI* hospital-acquired AKI, *CNY* Chinese yuan

### LOS and daily cost

The LOS and daily cost were similar in different patient groups categorized by age (Table 5). However, significant associations of AKI severity and LOS and daily cost during hospitalization were observed in the elderly patient group. In patients with AKI stage 3, the LOS was 19 days, and the daily cost was 3450 yuan, which was much higher than in patients with lower stages (Table 6).

### Renal outcome

Of note, different patient groups categorized by age had a similar proportion of patients requiring dialysis, whereas the renal recovery rate was much lower in the elderly patient group, especially in patients older than 80 years (33.8 %) (Table 5).

## Discussion

In this large, retrospective cohort study conducted in nine regional central hospitals across Northern, Central and Southern China, we have determined the incidence of AKI in Chinese elderly patients. By analyzing data from both young and elderly groups, we also illustrate risk factors and in-hospital outcomes of AKI in patients aged more than 65 years.

In our previous study, we determined that the frequency of SCr tests has a substantial impact on the detection rate of AKI [5]. By using a novel approach after adjusting for the frequency of SCr tests and other cofounders, we have estimated the incidence of AKI in the whole population at 11.6 %. The same method was used in the analysis of elderly patients. The adjusted incidence of AKI is 15.44 % in patients 65–80 years old and 22.22 % in patients older than 80 years, which is much higher than in younger groups. It was reported that the incidence rate of AKI is 14.8 % in Chinese very elderly patients (≥80 years) [12] and 8.6 % in patients older than 65 years who received contrast-enhanced CT [14], which is much lower than in our cohort because a large number of AKI patients were not detected by SCr measurements. Among the 167.5 million patients (data from National Health and Family Planning Commissions of the Peoples’ Republic of China) [17], we estimate approximately 8.4 million elderly patients suffered from AKI in 2013 in China, which is a huge medical burden.

In order to identify the contribution of different risk factors to AKI in elderly patients, PAF values were used in this study. In both HA-AKI and CA-AKI groups, preexisting CKD is a major risk factor for AKI. This may be explained by the susceptibility to kidney injury and greater incidence of comorbidity in patients with CKD. Indeed, another cohort including hospitalized patients at different CKD stage demonstrated that the risk of AKI increased with decreasing eGFR (8.9 % with eGFR ≥60 mL/min/1.73 m^2^vs 68.9 % with eGFR <30 mL/min/1.73 m^2^) [18]. In our previous study, we found urinary tract obstruction was an important risk factor for AKI [5]. The OR of urinary tract obstruction in this study was 1.67 in patients aged 18–64 years, 2.16 in patients aged 65–79 years and 2.75 in patients older than 80 years, suggesting that elderly patients with urinary tract obstruction were at higher risk for AKI. However, the PAF of urinary tract obstruction decreased when the patient population was getting older. This may be explained by the lower frequency of urinary tract obstruction in elderly patients. This highlights the use of ultrasound examination of urinary system in diagnosis of AKI in elderly patients. Of note, polypharmacy was very common in elderly patients [7]. Our study has shown that elderly patients have a high proportion of exposure to nephrotoxic drug, especially patients who use traditional Chinese medicines, which is consistent with previous study [12, 19, 20]. Although we could not identify the direct link between use of traditional Chinese medicines and AKI, more attention should be paid by physicians when prescribing traditional Chinese medicines to elderly patients.

In line with our previous study, we have demonstrated that in-hospital mortality, length of stay and daily cost in the elderly group are higher than in the younger group [21]. The mortality rate of AKI in our study (10.3 % in patients with age from 65 to 80 and 19.6 % in patients older than 80 years) is consistent with previous study [21]. In addition, the severity of AKI in elderly patients was associated with short-term outcomes and resource utilization. Compared with patients with other stages of AKI, elderly patients with Stage 3 AKI have lower recovery rates and higher mortality and daily cost, which indicate that those patients need special medical care in the clinical practice.

Even though this study included a large number of patients from multiple centers in China and a novel analytic approach was used, there are several limitations. First, estimated incidence rate is an approximation of the true AKI incidence because multiple or daily SCr tests are needed in order to increase detection rate of AKI according to KDIGO criteria. Second, urine output, another important factor of AKI detection, is not used in this study because of the lack of urinary data, which is also common in other studies on patients from ICU and non-ICU departments [20] However, this may lead to underdiagnosis of AKI [22]. Third, this study is retrospective research, and only in-hospital outcomes were analyzed. Long-term follow data are lacking. Fourthly, because this study lacks information on albuminuria and proper diagnosis of CKD 1 and 2, we could not analyze the data in patients with early stage CKD and albuminuria. And we exclude patients with a history of stages 4–5 CKD, maintenance dialysis or renal transplantation, which is similar to other studies [6, 23]. Therefore, only patients with eGFR from 30 to 60 ml/min are analyzed as patients with CKD in our study. Finally, information about drug induced AKI in this study is limited because prescription data are not complete in non-AKI patients and a uniform standard for identifying and grading the effect of nephrotoxic drugs is not available.

## Conclusion

In this large retrospective cohort study in China, we have determined the incidence of AKI is 15.44 % in patients 65–80 years old and 22.22 % in patients older than 80 years by adjusting SCr test frequency. Compared with young patients, older patients are more likely to develop AKI, and their outcomes are worse. Early detection and effective treatment of AKI in elderly patients should be the focus of future research.

## Abbreviations

95 % CI: 95 % confidence interval
AKI: Acute kidney injury
CA: Community-acquired
CAD: Coronary artery disease
CKD: Chronic kidney disease
CNY: Chinese yuan
EACH: Epidemiology of AKI in Chinese Hospitalized adults study
HA: Hospital-acquired
KDIGO: Kidney disease: improving global outcomes
LOS: Length of stay
NSAIDs: Nonsteroidal anti-inflammatory drugs
OR: Odds ratio
PAF: Population attributable fractions
SCr: Serum creatinine
WHO: World Health Organization
